# Maternal Satisfaction With Midwives’ Caregiving in Public Health Facilities: A Scoping Review

**DOI:** 10.7759/cureus.107858

**Published:** 2026-04-28

**Authors:** Dimitra Souflia, Athina A Samara, Michel Janho, Christina Messini, Maria Saridi, Evangelos C Fradelos, Aikaterini Toska

**Affiliations:** 1 Laboratory of Clinical Nursing, Department of Nursing, University of Thessaly, Larissa, GRC; 2 Department of Embryology, University of Thessaly, Larissa, GRC; 3 Department of Pharmacology, Faculty of Medicine, University of Thessaly, Larissa, GRC; 4 Department of Family and Community Medicine, General Hospital of Larissa, Larissa, GRC; 5 Department of Obstetrics and Gynecology, University Hospital of Larissa, University of Thessaly, Larissa, GRC; 6 Public Health and Adults Immunization Lab, Department of Nursing, University of Thessaly, Larissa, GRC; 7 Department of Nursing, University of Thessaly, Larissa, GRC

**Keywords:** antenatal care, delivery and labor, midwife care, obstetrics, satisfaction

## Abstract

Midwives play an important role in women’s childbirth-led care as they provide a protective role against maternal stress and foster deeper relationships. Midwives can significantly influence satisfaction levels, particularly when they provide consistent, high-quality, and empathetic support throughout labor and delivery, with potential positive implications on healthcare systems. This study aimed to review the current literature on indicators of maternal satisfaction with midwife care received during childbirth. A comprehensive search of the available literature regarding maternal satisfaction from midwives’ caregiving was conducted using the databases PubMed and Scopus. Cumulative evidence supports the idea that midwifery care is a primordial factor in maternal satisfaction. Midwives’ caregiving is pivotal in terms of maternal satisfaction from public health facilities. Given the plethora of contextual, cultural, and methodological parameters that influence maternal satisfaction, suitable and well-founded tools are needed to ensure continuous quality of care and to address current deficits in health systems. More high-quality studies need to be conducted in order to investigate the critical role of midwifery care during antenatal care.

## Introduction and background

Substantive progress in reducing maternal mortality and morbidity has been made in the past two decades. Specifically, recent data show a 40% reduction in maternal mortality since 2000. However, a staggering 260,000 women died in 2023 due to pregnancy-related pathologies. Maternal mortality ratios (MMRs) differ between countries. Sub-Saharan Africa contributes about 70% of the worldwide MMR mainly due to conflict and health system fragility [1]. On the other hand, a lower MMR is observed in Europe, but achieving excellent quality care in Europe can be challenging even in high-income settings [2]. For example, a four-fold variation in MMR was found between 8 European high-income countries that utilize surveillance systems permanently that use enhanced methods identifying, documenting, and reviewing maternal mortality [3]. Adversely, more than half of maternal and neonatal related preventable deaths in low and middle-income countries are associated with low quality in maternal care [4].

The World Health Organization (WHO) endorses the importance of maternal care as seen in several WHO articles [5-7]. This has been evident in the framework the WHO developed for maternal and newborn quality of care, where a key domain of care is centered around care experience and the importance of collecting the woman’s point of view [8]. Based on these principles, the WHO developed the ‘standards for improving the Quality of Care for mothers and newborns at the facility level [9]. These standards include over 300 quality measures centered around measuring quality of care comprehensively around the time of childbirth. Out of these 300 measures, women’s satisfaction with care is considered one that should be monitored and evaluated for identifying priorities of action that aim at improving the quality of care for both mothers and newborns [8]. These standards promote a person-centered philosophy that optimizes health as well as the general well-being of mothers and newborns alike [8]. Moreover, respectful and person-centered maternity care is directly linked to positive maternal outcomes, influencing care-seeking behaviors and maternal mental health according to recent evidence [10].

Midwives play an important role in women’s childbirth-led care. They provide a protective factor against stress for the mother as well as foster deeper relationships with her when all aspects of care are coordinated by the midwife [11,12]. Furthermore, they significantly influence satisfaction levels, particularly when they provide consistent, high-quality, and empathetic support throughout labor and delivery, with potential positive implications for healthcare systems [13]. Various prerequisites, however, need to be addressed to ensure this high level of care entails positive maternal experiences and improved health outcomes for both mothers and infants [14]. These primarily include an enabling environment where safe and effective care can be delivered, which extends beyond clinical competencies and allows midwives to fully implement their model of care [15-17].

Satisfaction is difficult to define as it is affected by a variety of factors, including maternal expectations, that may vary across women due to different contexts, such as their socioeconomic background, education level, and individual preferences, which are among the various elements that determine this level of satisfaction [18]. Maternal satisfaction refers to a mother's feelings of joy as a result of comparing a service provided in a health facility to her expectations and the ability of the services provided to meet her expectations, representing a crucial factor when choosing a health facility, compliance with services and follow-ups, and continuation of healthcare in effect, maternal satisfaction with midwife care is instrumental to the overall childbirth experience and can produce positive maternal outcomes [19]. The difference in maternal satisfaction from midwife care can be attributed to the quality of interaction, psychological support, and the establishment of mutual trust between mother and midwife, along with the well-being of the midwife, for it directly impacts the quality of care provided [20-22]. WHO recommendations highlighted dignity and respect, communication and autonomy, and supportive care during childbirth as key components of person-centered maternity care that should be provided for all mothers during labor and delivery, aiming to improve communication between health care providers and women to promote the utilization of care [23].

Therefore, this study aimed to review the current literature regarding indicators of maternal satisfaction with the midwife care received in public facilities around childbirth. To the best of our knowledge, the present study is the first study following a systematic approach of a scoping review analyzing published data regarding maternal satisfaction from midwives' care in public facilities. Public facilities were chosen as they reflect the caregiving in every socioeconomic background worldwide.

## Review

Methods

The present study was carried out in accordance with PRISMA extension for scoping reviews (PRISMA-ScR) [24].

A comprehensive search of the available literature regarding maternal satisfaction from midwives’ caregiving was carried out in the online databases Medline (PubMed) and Scopus. The last search date was 01/03/2026. The MeSH terms that were used were “satisfaction,” “midwife,” and “care.” Moreover, a manual search of related articles was conducted. 

All studies, including original data reporting maternal satisfaction from midwives’ caregiving in public health providing centers, whose outcomes could be retrieved and were reported in English, were considered eligible.

Exclusion criteria for the present studies were: 1) non-human studies, 2) outcomes of no interest, 3) not reported in English, 4) irretrievable outcome data, and 5) studies in the form of letters, editorials, expert opinion, or conference abstracts.

All eligible studies were submitted to rigorous quality and methodological assessment on the basis of the Cochrane’s risk of bias assessment tool [25]. Two independent researchers screened the identified data for eligibility. In cases of disagreement, a third senior investigator reviewed the data.

Results

**Figure 1:**
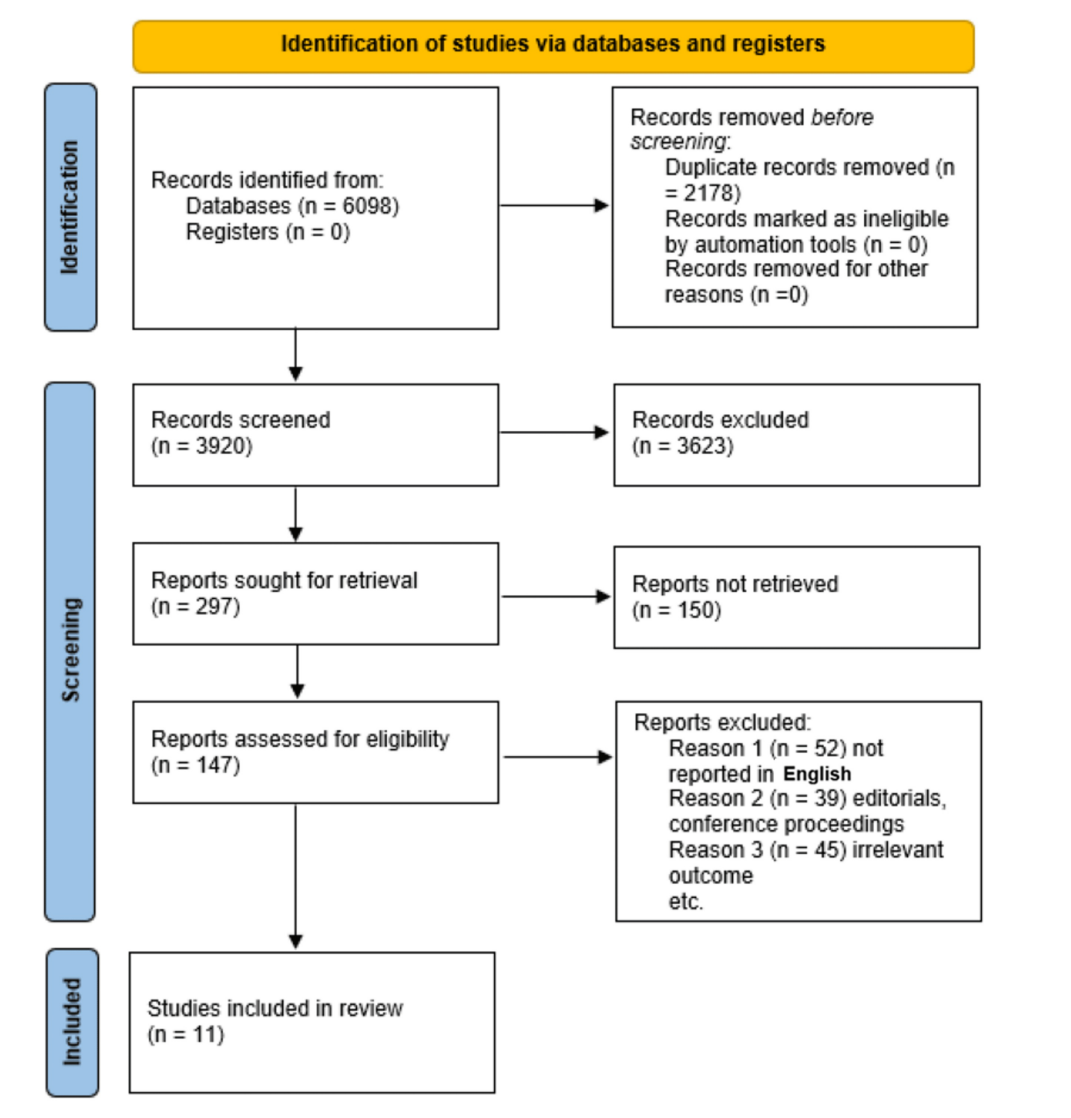
PRISMA flowchart PRISMA: Preferred Reporting Items for Systematic reviews and Meta-Analyses

In total, 11 studies were identified as eligible and included in our synthesis of evidence (Figure 1). Most of the data were from low-income countries where midwife care is the main course of caregiving.

In Table 1 are displayed the main results of the search of the available literature. The presence of a midwife is quintessential for the satisfaction of the mother during childbirth. Indeed, midwifery care is a primordial factor in maternal satisfaction, as exhibited by Harvey et al. [26], where it was found that the presence of an expert midwife providing clinical, emotional, and social support was associated with higher satisfaction than the care provided by doctors alone using the Labor and Delivery Satisfaction Index (LADSI), accompanied by a quick survey. Moreover, a systematic review of Macpherson et al. [27] included 24 studies that aimed to quantify maternal satisfaction from the care received by different healthcare professionals (gynecologists, obstetricians, nurses, and midwives) found that higher satisfaction was procured from the midwife's personalized care as well as childbirth within a family or a health facility setting [27]. Furthermore, this study also proposed that factors such as social health, professional health care, and health support are contributing to maternal health as they exhibited the highest weight in the analysis [28-30], thus promoting the need for specialized care provided by the midwife.

**Table 1:** Summary of evidence regarding satisfaction from midwifery care

Author	Study	Year	Country	Study Type	Questionnaires	Conclusions/Remarks
Harvey et al. [26]	Evaluation of satisfaction with midwifery care	2002	Canada	Comparative study	LADSI, ADLE, SSQ	Women experiencing low-risk pregnancies were more satisfied with care by midwives than with care provided by doctors. Satisfaction scores were high for both groups and may have been lower for women in the doctor group as a result of disappointment with caregiver assignment, as all women had sought midwifery care. The SSQ measures similar dimensions to the LADSI
Goberna-Tricas et al. [31]	Satisfaction with pregnancy and birth services: The quality of maternity care services as experienced by women	2010	Spain	Qualitative study		The mothers in this study were satisfied with health-care technology and viewed it as a source of security. Technology was indispensable to reducing the anxiety provoked by their perceived lack of confidence in their ability as mothers. During pregnancy and, especially, when giving birth, women believe that their feelings and values should be understood by professionals, from whom they seek empathy and personal commitment, not just information
Jha et al. [32]	Satisfaction with childbirth services provided in public health facilities: results from a cross-sectional survey among postnatal women in Chhattisgarh, India	2017	India	Prospective cross-sectional	WDEQ-B, EPDS, SMMS: Vaginal and Caesarian Births	Most women (VB 68.7%; CB 79.2%) were satisfied with the overall childbirth services received. Main differences in women giving Vaginal birth were due to public health facilities' shortages/the cesarean group is much smaller than the vaginal group
Mocumbi et al. [33]	Mothers’ satisfaction with care during facility-based childbirth: a cross-sectional survey in southern Mozambique	2019	Mozambique	Prospective cross-sectional		Μost of them were satisfied with the care during childbirth and would recommend a family member to deliver in the same facility. Mothers who gave birth in primary level facilities tended to be more satisfied than those who gave birth in hospitals, and the presence of a companion had a positive influence on the level of satisfaction, irrespective of age, education, and socioeconomic background
Astuti et al. [34]	Intrapartum care satisfaction at three levels of healthcare facilities in Jakarta	2019	Indonesia	Prospective cross-sectional	CEQ, Postpartum Support System Questionnaire, Family Coping Questionnaire, Satisfaction with Intrapartum Care Questionnaire	The pattern of satisfaction with intrapartum care did not differ significantly between the three levels of healthcare facilities
Hailemariam et al. [35]	Mother’s Satisfaction towards Childbirth Care at Public Health Centers in Bench-Maji Zone, Ethiopia: A Facility-Based Cross-Sectional Study	2020	Ethiopia	Prospective cross-sectional	Client satisfaction measuring instrument adapted from the Ethiopian Ministry of Health	The overall satisfaction of mothers with childbirth care in public health centers of Bench-Maji Zone is low when compared with other health centers in Ethiopia. Hence, understanding mothers’ expectations, assuring privacy, and enhancing antenatal care attendance might improve maternal satisfaction with childbirth care
Arrebola et al. [36]	Women’s satisfaction with childbirth and postpartum care and associated variables	2021	Spain	Longitudinal, observational study	STAI, COMFORTS	Eutocic delivery and skin-to-skin time with the newborn are associated with more satisfaction, whereas babies who are separated from their mothers and unmet expectations lead to less satisfaction. Anxiety doesn’t seem to play a part in lowering satisfaction
Kidane et al. [37]	Maternal satisfaction with delivery care services and associated factors at public hospitals in eastern Ethiopia	2022	Ethiopia	Prospective cross-sectional	Donabedian Quality Assessment Framework	The study revealed that four-fifths of mothers were satisfied with the delivery care services provided in public hospitals. Delivery through Cesarean section was associated with satisfaction
Esan et al. [38]	Assessment of satisfaction with delivery care among mothers in selected health care facilities in Ekiti State, Nigeria	2022	Nigeria	Descriptive cross-sectional study	WQBLS4	The majority (94.8%) of the respondents were satisfied with the delivery services provided at the facilities, while 14 (5.2%) expressed dissatisfaction. A significant association exists between respondents’ level of education and maternal satisfaction with delivery care (p < 0.05)
Tolesa et al. [39]	Maternal satisfaction with intrapartum care and associated factors among mothers who gave birth in public hospitals of the Southwest Shewa Zone, Ethiopia	2023	Ethiopia	Prospective cross-sectional		More than half of mothers (59.3%) were satisfied with the overall intrapartum care they received. Vaginal delivery, labor duration <12 hours, and waiting time< 15 minutes were associated with maternal satisfaction with intrapartum care
Ratislavová et al. [40]	Measuring women’s satisfaction with childbirth: a literature review of measurement properties	2024	Czech Republic	Systematic review for the use of questionnaires		Thorough testing of tools measuring women’s satisfaction with childbirth, and adapting them to cultural and social contexts, is still essential. Valid and reliable questionnaires must be available for midwives in practice, for use in research, to inform clinical practice, and for the results to help develop the services offered.

Also, a sense of emotional security can be reinforced via empathetic care by understanding women’s feelings and values, especially during childbirth, as well as the presence of advanced healthcare technology to reduce maternal anxiety [31]. On the other hand, results from a cross-sectional survey conducted in India, including postpartum women, reported that while most women were satisfied with the services provided during childbirth, albeit with shortages in the public health care system, specifically vacant midwife positions, influenced negative experiences in vaginal births [32].

Many other factors can also play a part in maternal satisfaction, and these can differ according to contextual and cultural factors. A prospective cross-sectional study in Mozambique reported higher maternal satisfaction from births in primary health centers than hospitals [33]. The presence of birth companions further improved these satisfaction levels. Meanwhile, a similar prospective cross-sectional study in Indonesia showed no significant difference in satisfaction across three different health facility levels [34].

Some studies, however, reported mixed results. Specifically, a prospective cross-sectional study in Ethiopia found lower satisfaction levels in mothers in certain regions in the country, while over half of the mothers recruited were satisfied with the care provided (62.5%). Factors influencing satisfaction levels included privacy and antenatal attendance [35]. Similar studies held in the same setting found that caesarian delivery had a positive effect on maternal satisfaction, while long duration of labor and waiting times contributed negatively to satisfaction levels [36,37].

Other non-clinical factors should also be considered. A longitudinal study in Spain revealed that unmet expectations reduce maternal satisfaction regardless of anxiety, whilst skin-to-skin contact increased satisfaction levels [38]. On the other hand, education level has been reported as a key parameter in satisfaction [39,40].

Discussion

Our study tried to investigate the impact of midwives’ caregiving during the antenatal period. Promoting patients’ satisfaction is crucial in reducing patient negative feelings such as anxiety, improving treatment adherence, and promoting health quality [41]. Increasing patients’ satisfaction level with health services has long-term benefits for both the community and patients. Based on our results, more and more cumulative data have proved the positive impact of midwives’ care during pregnancy and labor. We need to underline the importance of expanding midwifery services to further improve maternal and neonatal health outcomes and overall patient satisfaction.

The choice of questionnaire has to be considered carefully. The questionnaire must demonstrate the capacity to measure women’s satisfaction accurately and tailor to different cultural contexts. In the review by Ratislavová et al, the Childbirth Experience Questionnaire (CEQ/CEQ2) and the Birth Satisfaction Scale-Revised (BSS-R) are the most prevalent questionnaires in the literature [40]. The BSS-R is a fast and simple 10-component questionnaire in comparison to the CEQ/CEQ2 which is composed of 25 items. Both questionnaires have demonstrated excellent psychometric properties in multiple studies [42,43]. The CEQ/CEQ2 also excels in its ability to differentiate satisfaction based on delivery method [44]. However, the International Consortium for Health Outcomes Measurement committee recommends the BSS-R as the primary tool to quantify women’s childbirth experiences [45,46], and as of 2020 has been used worldwide in 39 countries across 134 sites [47].

Variations in satisfaction can be found in different areas of the same country. Ethiopia is a good example, given the plethora of studies exploring women’s satisfaction with healthcare during childbirth [39,47,48]. Differences in satisfaction amongst these studies are speculated to concern the research environment and the facilities available for healthcare. The presence of the healthcare provider, i.e., the midwife, as well as the availability of doctors and midwives, had the highest satisfaction scores [37], emphasizing the pivotal role of the midwife in childbirth and ultimately contributing to women’s satisfaction.

It is argued that better maternal satisfaction outcomes occur at primary healthcare settings in comparison to hospitals. Indeed, a 2019 prospective cross-sectional study reported higher maternal satisfaction at the primary healthcare setting than at hospitals [35]. Other studies in different cultural contexts confirmed this finding [49-51], and reason that overcrowded hospitals could be one of the factors for higher satisfaction in smaller facilities. Conversely, intrapartum satisfaction remained the same across all levels of healthcare in the cross-sectional study by Astuti et al. [34]. The presence of the midwife and the support they provide across all levels of healthcare could be a possible explanation for the mixed results.

Mahmood et al. [52] concluded that women who received antenatal care (ANC) services from facilities with midwives reported significantly higher satisfaction ratings compared with those who received ANC in settings without midwifery support. Except for midwives’ services, informal education among women, follow-up in ANC, a planned delivery in health providing centers, respecting women's privacy, offering service free of charge, time to be seen by the healthcare providers, and duration of labor were associated with women's satisfaction rates [53]. In Serbian public services, analyzing data from 34,431 questionnaires, the highest average satisfaction score was reported for the overall participation of midwives during delivery [54].

Finally, given the plethora of contextual, cultural, and methodological parameters that influence maternal satisfaction, suitable and well-founded tools are needed to ensure continuous quality of care and to address current deficits in health systems. Moreover, as the majority of evidence came from low-income countries, the generalizability of our results remains low. More high-quality studies need to be conducted to investigate the critical role of midwifery care during ANC. Data from the Western world is also essential to better understand the parameters of maternal perception of satisfaction with the ANC.

## Conclusions

Midwives’ caregiving is pivotal in terms of maternal satisfaction from public health facilities. Given the plethora of contextual, cultural, and methodological parameters that influence maternal satisfaction, suitable and well-founded tools are needed to ensure continuous quality of care and to address current deficits in health systems. More high-quality studies need to be conducted in order to investigate the critical role of midwifery care during ANC.
